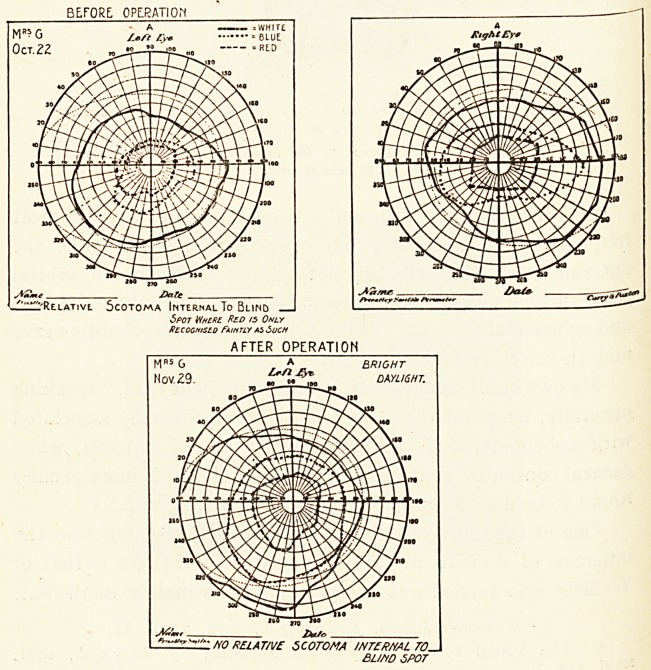# Specialism and the Medical Curriculum, Mainly in Reference to Otology, Rhinology and Laryngology

**Published:** 1913-12

**Authors:** P. Watson-Williams

**Affiliations:** Lecturer on Otology, Rhinology, and Laryngology at the University of Bristol; Hon. Aurist and Laryngologist, Bristol Royal Infirmary.


*"Scire est nescire, nisi id me*
*Scire alius sciret."*

DECEMBER, 1913.

### SPECIALISM AND THE MEDICAL CURRICULUM, MAINLY IN REFERENCE TO OTOLOGY, RHINOLOGY AND LARYNGOLOGY.

**The Presidential Address, delivered on October 8th, 1913, at the opening of the Fortieth Session of the Bristol Medico-Chirurgical Society.**

BY

P. Watson-Williams, M.D. Lond.,

*Lecturer on Otology, Rhinology, and Laryngology at the University of Bristol; Hon. Aurist and Laryngologist, Bristol Royal Infirmary.*

---

The subject of my remarks, which I offer in thanks to this Society for having honoured me in electing me as its President, is a different aspect of the subject of a Presidential Address at the Laryngological Section of the Royal Society of Medicine in 1910, and again of my opening of a discussion at the Annual Meeting of the British Medical Association at Liverpool in 1912. Then I was addressing specialists on the "Education of the Specialist"; now, under the roof of our University, I plead the cause of the undergraduate and postgraduate student.

515646

It is a mistake as to suppose, many of us do, that specialism in medicine or surgery is a modern innovation. In the Ebers papyrus are many allusions to various special regional affections, *e.g.* "When thou findest a growth on the throat of a patient . . . containing matter . . . and thou findest its stock raised like a wart, know that the matter moves within it." We find that the practice of surgery was distinguished from medicine even earlier than the thirteenth century B.C., and it is recorded that in Egypt each physician treated a single disorder and no more, some devoting themselves to the eye, others to the teeth (they made artificial teeth, and some have asserted that they have found traces of gold stoppings in the mummies), and others, again, devoted themselves to disorders of the intestines, while yet others were obstetric physicians called in by midwives to the difficult cases.

And we learn from the Ebers papyrus that in the thirteenth century B.C. (*i.e.* about 3,200 years ago) patients applying for relief to the medical temple at Thebes had to state their complaint, and that it was left to the principal of the medical staff to send the specialist best suited for the case. It would appear that only a few of the students remained to the end of their medical course at Thebes, the most gifted being sent to the celebrated faculty of Heliopolis, whence they returned to Thebes, where they became physicians to the court, and, attached to some priestly college, were consulted in serious cases.

Now in Great Britain, and later also in Ireland, the field of medical science was long occupied by rival schools of medicine and of surgery; hence perhaps arose our academical distinction between medicine and surgery peculiar to this country, for in practically every European nation except our own the degree of M.D. serves for all physicians, surgeons and specialists alike, their subsequent postgraduate differentiation is clinical, and not academical and dependent on the passing of certain examinations, as is the case with us. We attach such importance to the acquirement of innumerable useless minutiæ, that research work is in consequence comparatively rare, and is put, so to speak, under a glass case, where it is admired but seldom touched by the common herd. Is it not better to train a student to think for himself and to investigate, instead of loading his mind with facts which for the most part he is not even intended to remember after he has passed the rubicon? What a misdirection of time and energy when we compel a young university graduate in medicine and surgery, with opportunities and ability for research work, to go back to the kind of anatomy required for our highest surgical diplomas!

This bowing of the knee to the Baal of examinations, which is characteristic of this country, coupled with the traditional restricted import of the academic labels of "physician" or "surgeon," has resulted in the tendency to regard specialities as accretions to the medical art and science, whereas they are more truly an intensive cultivation of relatively neglected areas in the common field.

Great contributions to the recent progress of medical work have mostly come from those who have had large opportunities for practical experience in relatively restricted fields of observation; and as a hospital clinic—medical, surgical, or special—affords peculiar opportunities for scientific investigations and advancement of knowledge, those who hold such offices should regard their posts as trusts, and as far as possible render themselves competent to make such opportunities fruitful and help one another in so doing, not for their own advancement, but for the credit of their school, for the benefit of the profession, and for the sake of humanity at large. The far-reaching later developments of the science and art of medicine have compelled us to realise that to be highly productive in the best sense we must revert to the very natural division of fields for research that obtained in Egypt so many thousand years ago.

When I was a student anatomy, physiology, and pathology were well taught by surgeons or physicians, one of the obstetric professors was, and only one of the clinical surgeons was not, a general practitioner; there was no pure ophthalmic surgeon and not one laryngologist in Bristol. But specialisation is now regarded as essential for those who are entrusted with teaching of these subjects, and in the truest sense all have become specialists, for the pure surgeon or internal physician is as distinctly a specialist as the obstetrician, ophthalmologist, laryngologist or aurist, and not seldom more so. If, then, I use the word specialist in any further remarks in the more usually accepted sense, I at least recognise the folly of trying to separate sheep from goats in the medical flock. As for myself, if you will pardon a personal note, I was quite determined never to become a specialist in diseases of the ear, nose and throat, when with a view to becoming a physician and specialising in the organs of respiration I published a short work on the upper respiratory tract, intended as a prelude and introduction to a second volume on the lungs and pleura. Fortunately or unfortunately, my first volume seemed to meet a want, and moreover served to make me realise how little I knew of a most interesting and hardly explored territory, which lured me on till it secured my undivided attentions. My colleagues at the Royal Infirmary were good enough to respond to my suggestion in 1889 that I should initiate a clinical throat department, and it is owing to them that it has fallen to my lot to share in the development of modern laryngology. Up to that date the special equipment for diseases of the throat and nose consisted of a mottled laryngoscopic mirror, one Thudicum speculum, a Mackenzie tonsillotome, and a pair of Loewenberg forceps, the latter rarely used, as adenoids were almost unknown, and when diagnosed were mostly scraped with a finger-nail. In 1887 we began to hear of intubation as an alternative to tracheotomy, and as a Resident at the Royal Infirmary I inserted a tube in a diphtheritic larynx, I believe the first case in this country, and great was my relief when the urgent dyspnœa almost instantly gave place to calm breathing. Since that time laryngology has been enriched by the acceptance of Semon's work on the innervation of the larynx and the early diagnosis of malignant growths of the larynx and their successful operative treatment. Œsophagoscopy and bronchoscopy have been simplified and rendered practicable by Killian, Chevalier Jackson, Brünings and Hill, to mention only a few of the laryngologists to whom we owe these advances, and which we in Bristol have utilised in the throat department since 1904.

The relative ease with which a bronchoscope can be passed has been demonstrated at the meetings of this Society. Nothing could more clearly demonstrate its far-reaching achievements than the case of a patient in whom a nail had become implanted for a long period in a second division bronchus, when Killian was able to dilate the cicatricial contraction, extract the nail and implant a gold collar, which the patient wore to keep that part of the bronchial tube patent.

In rhinology the pathology, diagnosis and treatment have been practically a new field opened up, and in otology Bárány's work on the diagnosis and Lake's on the operative treatment of labyrinthine disease have led the way, and made possible the relief in a group of affections which before then were almost untouched by clinicians.

To mention only these few of the many directions of advance must suffice to show that the territory is by no means narrow. No speciality can thrive and progress in sound directions unless its votaries return constantly to drink at the common fount of medical knowledge. So likewise its culture never fails to enrich the common ground in almost every corner of the medical field. As an example in point I invite you to glance at just one small corner of rhinology, and that only in so far as it bears on the work of other departments.

**The nose in relation to disease of the eye** is a subject that has of late years engaged the attention of many rhinologists and ophthalmologists, so that the literature of the relationship between diseases of the nose and eye is already very extensive, but many debatable points remain to be elucidated. Nor is it by any means a new subject, for Berger devotes seven pages to it in his treatise *Les Maladies Des Yeux dans leurs Rapports avec la pathologie générale* (Paris, 1892). But rhinology, then in its infancy, has advanced, and with improved clinical methods of investigation, and a fuller knowledge of the regional anatomy, many eye conditions may be proved to have their source in the nose, where formerly such causes were seldom looked for or even suspected.

It must suffice to direct attention to the four excellent preparations by Onodi, which by his courtesy I am enabled to introduce to illustrate some of the more important anatomical relationships of the orbit and nasal passages and sinuses.

FIG. 1.

SECTION BEHIND THE FIRST MOLAR TOOTH.

The right sphenoidal sinus is fairly normal, but the left is quite small and almost central, being encroached on by the backward extension of a large posterior ethmoidal cell. The right optic canal traverses the upper and outer wall of the right sphenoidal sinus, but the left sphenoidal sinus is not in relation with the left optic canal, which passes across the upper and outer part of the left posterior ethmoidal cell.

1. Right optic nerve.

2. Left optic nerve.

3. Left posterior ethmoidal cell.

5. Superior turbinal.

6. Middle turbinal.

7. Left maxillary antrum.

8. Left inferior turbinal.

9. Right maxillary antrum.

10. Left sphenoidal sinus.

11. Right sphenoidal sinus.

ONODI.

FIG. 2.

A HEAD WITH THE ORBITAL WALLS REMOVED.

The preparation shows the relations of the roof, inner wall and floor, to the frontal lobe and optic nerve and nasal accessory sinuses.

1. Recessus orbitalis.

2. Frontal sinus.

3. Nasal cavity.

4. Maxillary sinus.

5. Sphenoidal sinus.

6. Internal carotid artery.

7. Optic nerve.

8. Frontal lobe.

9. Dura Mater.

× × Anterior and posterior ethmoidal cells.

ONODI.

Berger in 604 cases of orbital inflammation found that 407 were due to nasal accessory sinus disease; while Birch-Hirschfeld, from an analysis of the records of the Leipzig eye clinic, showed that out of 684 cases of orbital inflammation no less than 409, or 59.8 per cent., were due to sinus inflammation, and further states that if an examination of the nasal sinuses had been made in every case the percentage would have been higher still.

I do not intend to discuss the accuracy of these figures cited from such distinguished authorities in ophthalmology, though I believe that in Bristol eye clinics we should not find these figures accepted without comment. But there is no longer any doubt as to the necessity for rhinology depending on the collaboration of the ophthalmologist in a very large percentage of sinus cases.

I pass over all the eye complications due to neoplasms, benign or malignant, originating in the nose or accessory sinuses, and those due to mucoceles, these as a rule being mainly displacements of the bulbus, at least in the earlier stages, and I will only allude very briefly to the orbito-ocular manifestations of nasal sinusitis. The normal nasal mucous membrane is sterile, when it becomes infected the resulting inflammatory reaction may prevent the infecting organisms reaching further than the submucosa, and after losing more or less of the ciliated epithelum it usually recovers.

With a more virulent infection the deep periosteal layer is infected, and an acute, subacute or chronic infective periostitis may remain, the case becoming one of chronic sinusitis. The mucosa becomes œdematous, and consequently the small apertures by which the sinuses communicate with the nasal passages are much occluded with retention of secretions, under more or less pressure. If the efferent lymphatic vessels in the deeper layers of the mucosa become permanently blocked, there is the formation of œdematous polypus (as I described in my Long Fox Lecture). With polypus formation either in an accessory sinus or its aperture, or in the nasal mucosa near it, the examination of the infected periosteum often shows evidence of an underlying rarefying and formative osteitis, and while spicules of new bone may be produced in other directions, the bone is softened or absorbed.

Such processes tend to involve the walls of the infected sinus in all directions. Take the case of an ethmoidal cell with its bony wall alone dividing the nasal passages from the orbital cavity, so thin that the inner orbital wall corresponding to the ethmoidal cells is called the lamina papyracea. We see how readily absorption of the thin wall may result in a perforation, a ready portal for infection of the orbital periosteum, while the frequent existence of congenital dehiscences often provides a ready-made way open for an infective invasion taking place before there is time for a perforation of the bony wall to be produced pathologically. However, the lymphatic and venous vascular communications normally existing between the sinus mucosa and the orbital periosteum are even more frequently the route for infection spreading from the nose to the eye, and although fortunately in only a small percentage does such infection result in orbital subperiosteal abscess or orbital cellulitis or phlegmon, in a very large number there are slighter evidences in the form of fugacious erythema or œdema of the forehead, eyelids or neighbouring tissues, swelling of the lids, conjunctival injection, chemosis, etc. Frequently errors of refraction develop either from the general neurasthenia result or from the direct toxic infection of the musculature of accommodation. Spectacles will correct the refractive errors, but the relief is relative and incomplete if the essential cause of trouble is untouched.

The maxillary antrum seldom causes an orbital phlegmon, the cavity being large and the roof least exposed to risks of perforation, but it often causes errors of refraction and weakness of vision, the "readily tired eye." Carious teeth may cause an orbital abscess, and in young persons, as Parinaud has explained, the venous and lymphatic channels of the unerupted tooth-buds form the route by which the infective organisms gain entrance to the orbit from the carious teeth. Two examples have been brought to my notice by our colleague, Mr. Fawn. These cases are prone to be very virulent and even fatal in event. One of Fawn's patients five years previously had iritis in the left eye, which became cured very quickly after the removal of a dead suppurating tooth, but on this later occasion she had marked iritis in the left eye, which grew steadily worse under the usual treatment for iritis, but rapidly subsided after the extraction of a decayed upper molar, both upper second pre-molars and some incisor pivots, all in a bad state. The second case which also developed irido-cyclitis not yielding to routine treatment, was rapidly cured after the extraction of diseased teeth.

The frontal sinuses, unless a perforation of the floor occurs, usually influences the eye through the fronto-ethmoidal infection that is nearly always present, either as cause or result of frontal sinus infections.

A case of cerebro-spinal rhinorrhœa associated with right frontal sinus and antral suppuration and nasal polypus is here illustrated, as it affords a good example of anatomical irregularity leading to fatal complications. The anatomical opening in the dura mater resulted in a fatal meningeal infection by the frontal sinus discharges. The case is fully reported elsewhere.[1]

FIG. 3.

*Right eye displaced downwards and outwards by retention of cerebro-spinal fluid in the orbit above and behind right inner canthus.*

[1] *Proc. Roy. Soc. Med. Lond.*, 1913, vii. 2.

FIG. 4.

1. *The opening in the dura mater through which it is presumed the cerebro-spinal fluid escaped into the right nasal passage.*
2. *The dark area corresponds to the large dehiscence in the bone of the orbital roof.*

It is the ethmoidal cells and sphenoidal sinuses that are most frequently the cause of orbito-ocular lesions, and while the anterior ethmoidal cells are not seldom the cause of orbital subperiosteal abscess or phlegmon, the posterior ethmoidal cells and sphenoidal sinus are the most constant source of optic nerve and therefore retinal affections.

My own cases seem to warrant the statement that, "speaking generally, temporal visual field contraction is usually associated with sphenoidal and posterior ethmoidal cell sinusitis, while general concentric contraction of the visual field is more usually found to be due to suppuration in the anterior group." [1]

One of the most valuable of many recent researches on the influence of sinusitis in causing visual field changes is that of Wallis, [2] who carried out his investigations mainly in the ear,

[1] Watson-Williams, *J. Laryngol.*, 1912, xxvii. 18.

[2] "The Visual Fields in Sphenoid Sinusitis," *J. Laryngol.*, 1911, xxvi. 242.

nose and throat clinic of our Royal Infirmary, and also at the Eye Hospital.

An example in point occurred in the wife of a medical man who had complained of supraorbital headaches for nearly three years, and of a sense of stoppage, and a bad smell and taste "coming from the back of the nose." The most careful examination of the nose failed to reveal any purulent secretion except a small amount of muco-pus in the left olfactory fissure, and syringing out the maxillary antra only yielded a few flocculi from the left antrum. Exploration and lavage of the sphenoidal sinuses also gave no definite result, beyond a small quantity of mucus from the left sinus. Dr. Leighton Davies examined and reported on the vision, "There is a slight but

BEFORE OPERATION

RELATIVE SCOTOMA INTERNAL TO BLIND SPOT WHERE RED IS ONLY RECOGNISED FAINTLY AS SUCH

AFTER OPERATION

NO RELATIVE SCOTOMA INTERNAL TO BLIND SPOT

unimportant contraction of the right field, but the left shows a distinct limitation of the visual field on the temporal side, and a small relative scotoma to red, internal to the blind spot. This had been observed several times, and I think it would have gone on to a complete scotoma to red." One month after I had performed a left intranasal antral operation with free removal of the anterior wall of the sphenoidal sinus, followed by lavage, the ophthalmic report was that "the left visual field showed marked improvement in the field for white as well as blue, and the disappearance of the relative scotoma. This shows, I think, quite clearly the need for radical operation in order to stop the progress of an early retrobulbar neuritis."

When orbital subperiosteal abscess, or orbital phlegmon, is suspected or diagnosed as due to nasal sinus disease, one should inspect the corresponding orbital wall, by an external incision and raising the orbital periosteum, so that one can see the bone, note any patch of softening, or any perforation. Even if deep-seated orbital cellulitis has developed one can by proceeding subperiosteally evacuate any subperiosteal abscess, and if necessary incise the orbital periosteum so as to drain or make applications to the orbital phlegmon.

As an example of eye complications from aural disease, I may refer to paralysis of the sixth nerve, which occurs most frequently following acute infection, and there are at least fifty-five cases on record, while I have had one case myself. Probably the infective inflammation extends forwards to cellular spaces on the apex of the petrous part of the temporal bone, through communications from the apical cells to the carotid sheath, or else by way of the cells around the Eustachian orifice and floor of the tympanum, and from thence to the fibrous sheath of the sixth nerve in Dorello's space.

But the physician, too, is often brought into direct relationship with the accessory sinus. Very many cases of so-called migraine are in reality examples of nasal sinusitis, and recurrent headache is as frequently due to similar nasal conditions as to errors of refraction. Quite recently a boy was admitted at the Royal Infirmary semi-conscious and believed to be a case of tubercular meningitis, but *post-mortem* it was found to be meningitis resulting from ethmoidal cell suppuration. In another case that occurred some years ago a girl was unconscious with symptoms of meningitis, but she had marked exophthalmos with turgid œdematous lids. A diagnosis of sphenoidal sinusitis with resulting cavernous sinus-thrombosis was diagnosed and confirmed *post-mortem*. Unfortunately she was seen too late for treatment to be of any use.

To even touch on the very numerous conditions in which a simple laryngoscopic examination throws light on bulbar lesions, pulmonary affections, aneurysms, or intrathoracic tumours, or a nasal inspection reveals the cause of recurrent bronchitis, hoarseness, dyspepsia, mental depression, and often, very often, mental aberration and suicidal impulses, would occupy too much time. But there are many fruitful fields for investigation still open, *e.g.* the nose as a route of infection in meningitis, *e.g.* cerebrospinal meningitis, nasal suppuration as a cause of appendicitis, rheumatoid arthritis, and so forth.

The University of St. Andrews, the National University of Ireland, and the Conjoint Board in Ireland of the Royal Colleges of Physicians and Surgeons are, I believe, the only corporations where such a definite clinical course is obligatory, *as well as examination by specialists*. Edinburgh, one of the most advanced centres as regards our speciality, with its splendidly organised teaching clinic, second to none in the United Kingdom, allows its candidates for graduation at the university a choice between pediatrics and oto-laryngology. One may well ask why both are not required.

Several universities in this country do insist on students attending a special clinic; others do not even go this far. But unless modern methods of professional examinations are to be applied to the special branches, the student is practically invited, and often compelled, to confine his serious attention to those subjects which help him to graduate, for the obvious reason that he has to satisfy examiners, and usually feels that he must devote his attention to the subjects on which he will be marked. Students should be both trained and examined by teachers who possess as thorough knowledge of otology and laryngology as we expect of the teachers and examiners in medicine, surgery, or gynæcology.

Special subjects should always be separately represented on the faculties of the universities and teaching schools by the heads of departments concerned. In our own University neither ophthalmology nor oto-laryngology are represented as such on the Medical Board, and when the clinical side of a medical school is in advance of the academical, the latter loses due perspective and proportion.

Is it not deplorable that even yet medical practitioners are frequently sent forth as qualified men without adequate *elementary* training in diseases of the nose, throat and ear, despite the fact that the curriculum has been extended for the express purpose of meeting this well-known defect ?

In the Dominion of Canada the universities are more progressive, for the various provincial licensing boards not only demand attendance upon the subject during the student's career at college, but also have an examination upon them for the licence to practice.

It is not quite fair to the student to insist on a paper acquaintance with a large number of surgical procedures which he can hardly ever utilise, and send him out into practice to lament his inability to deal with cases which come before him almost daily.

I cannot help feeling that some of the student's time which is now taken up in acquiring book knowledge of rare and difficult operations would be more usefully employed in gaining further acquaintance with departments of practice that they will find valuable in whatever direction their postgraduate work leads them, and on which they now often are completely ignorant on the day they are "qualified."

Again, it is small consolation to a practitioner to realise that his eighteen months or more dissecting and learning anatomy enables him to distinguish between the right and left pisiform though he cannot differentiate ordinary catarrhal from nerve deafness, or look into a nose and see whether an obstruction is a polypus or a congested turbinate ; or to examine a larynx and recognise a paralysed vocal cord or a tuberculous ulcer.

## Figures and Tables

**Fig. 1. f1:**
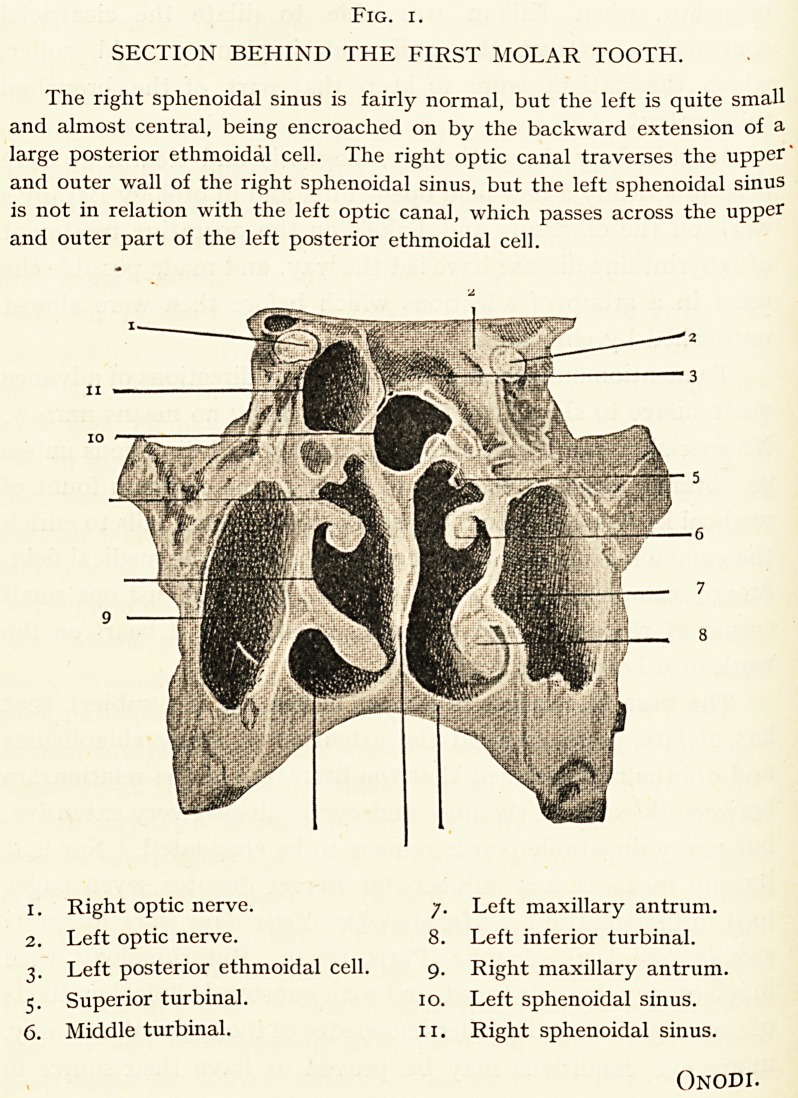


**Fig. 2. f2:**
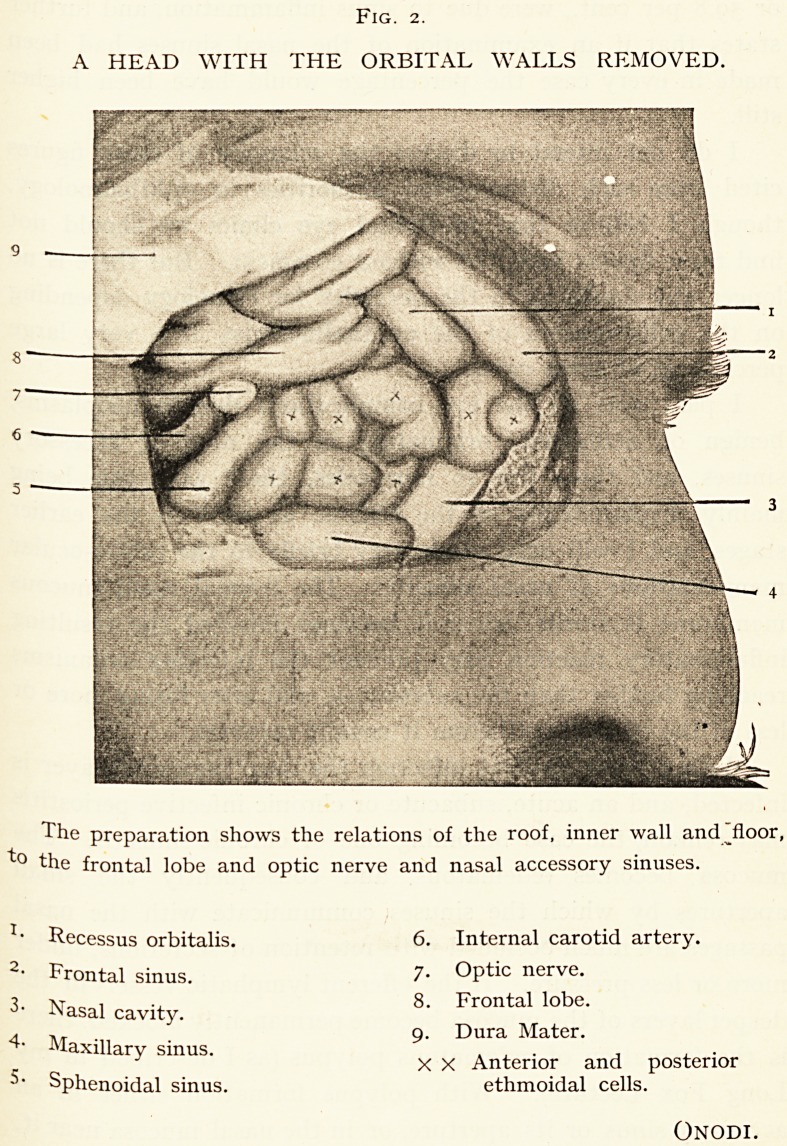


**Fig. 3 f3:**
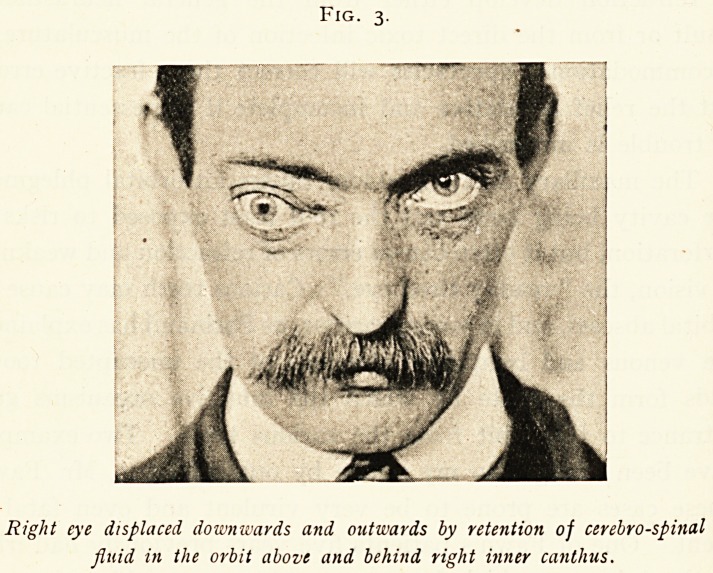


**Fig. 4. f4:**
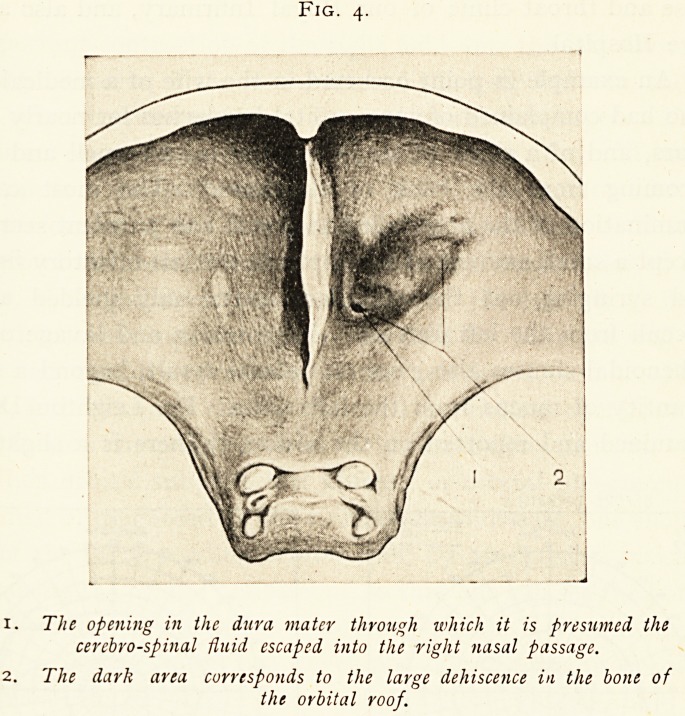


**Figure f5:**